# Associations between antibiotic exposure intensity, intestinal microbiome perturbations, and outcomes in premature neonates with bacteremia

**DOI:** 10.1038/s41372-025-02330-0

**Published:** 2025-06-09

**Authors:** Hope Hendricks, Shani Israel, Jörn-Hendrik Weitkamp, Suman Pakala, Seesandra Rajagopala, Ritu Banerjee

**Affiliations:** 1 Division of Pediatric Infectious Diseases, Department of Pediatrics, Duke University Medical Center, Durham, NC USA; 2 Department of Psychology and Human Development, Peabody College, Vanderbilt University, Nashville, TN USA; 3 Division of Neonatology, Department of Pediatrics, Vanderbilt University Medical Center, Nashville, TN USA; 4 Division of Infectious Diseases, Department of Medicine, Vanderbilt University Medical Center, Nashville, TN USA; 5 Division of Pediatric Infectious Diseases, Department of Pediatrics, Vanderbilt University Medical Center, Nashville, TN USA

**Keywords:** Translational research, Bacterial infection

## Abstract

**Background:**

Neonatal microbiome dysbiosis is associated with infectious complications.

**Methods:**

Prospective weekly stools were collected over 1 year from hospitalized preterm infants with birthweight ≤2000 g and postnatal age (PNA) ≤2 months. Neonates with bacteremia (cases) were matched to uninfected controls. Stools were analyzed using whole metagenome sequencing. Intensity of antibiotic exposure was compared using an Antibiotic Spectrum Index (ASI).

**Results:**

We analyzed 398 stools from 40 cases and 39 controls. Cases had lower α diversity beyond 4 weeks PNA. Cases with subsequent infections after index bacteremia had persistently lower α diversity, while cases without subsequent infections demonstrated recovery of microbiome diversity. Compared to controls, cases had greater ASI at multiple timepoints, higher *Enterococcus* spp. and lower anaerobe abundance.

**Conclusions:**

Compared to controls, premature neonates with bacteremia had intestinal microbiomes with lower α diversity, higher *Enterococcus* spp. and lower anaerobe abundance. These changes were associated with recurrent infectious complications.

## Introduction

Premature and low birthweight infants have high risk for infectious complications, including necrotizing enterocolitis (NEC), sepsis, and meningitis, and many receive empiric antibiotics at birth [1]. While antibiotics can be lifesaving for neonates with serious bacterial infections, increasing evidence demonstrates that antibiotic exposure significantly disrupts normal infant gut microbiome maturation and diversification [2, 3]. Some hospitalized neonates receive antibiotics for a few days after birth, whereas many others receive repeated or prolonged courses during their neonatal intensive care unit (NICU) admission, amounting to substantial cumulative exposure [1, 2].

The link between antibiotic exposure and microbiome disturbance is well-described, including associations between broad-spectrum antibiotics and reduced species diversity, increased prevalence of antibiotic resistance genes (ARGs), and predominance of pathogenic bacterial species in the microbiota [4]. One study examining the stool of 72 antibiotic-exposed term and preterm infants found anaerobic spp. abundance decreased with each additional day of antibiotic exposure, emphasizing that antibiotic exposure is not benign and that differing degrees of exposure ought to be measurable [5]. A growing body of literature associates microbiome disruption in neonates and other high risk populations with important diagnoses including NEC [6], late-onset neonatal sepsis [7], and graft-versus-host disease (GvHD) [8, 9]. A recently published double blinded trial by Getanda et al. found that intrapartum azithromycin use in mothers was significantly associated with lower *E. coli* and higher *Klebsiella pneumoniae* prevalence in the rectal swabs of their infants at 6 and 28 days of life [10]. That the same alterations were not appreciated in the mothers’ samples supports the notion that the developing and immature microbiome of neonates is particularly vulnerable to disruption following antibiotic exposure.

Despite many associations, establishing a causal link between antibiotic exposure, intestinal dysbiosis, and increased morbidity in neonates has been challenged by multiple confounders (e.g., diet, gestational age (GA), maternal history, mode of delivery). Two recent randomized controlled trials treated neonates at low or moderate sepsis risk with either empiric ampicillin and gentamicin for 48 h after birth or no antibiotics, and neither study found significant differences in clinical outcomes or microbiome diversity between intervention and placebo groups [11–13]. However, these studies of primarily healthy, late preterm infants examined short courses of relatively narrow spectrum antibiotics (ampicillin and gentamicin) and are not generalizable to a broader NICU population which includes complex and critically ill infants with extensive antibiotic exposures.

Other research has attempted to identify specific microbiome signatures associated with disease which might be clinically actionable. Some studies indicate that increased abundance of *Enterococcus* spp. in the gut flora may be a key driver of pro-inflammatory processes that increase patient morbidity. Among adult allo-hematopoietic cell transplantation (HCT) patients, high *Enterococcus* spp. abundance in the stool following HCT was associated with significantly reduced survival and with GvHD-related mortality [9]. Schwartz et al. reported that among 19 preterm infants with bloodstream infection, *Enterococcus* spp. abundance differed significantly by receipt of ampicillin, gentamicin or vancomycin in the prior 10 days [14].

Few studies quantify comprehensive antibiotic exposures over time and at the patient level, or associate such measures with concomitant microbiome changes. Recently proposed antibiotic intensity scores incorporate total days of antibiotic therapy with spectrum of pathogen coverage but have not yet been widely used in research or clinical practice [15–17]. One such score, the antibiotic spectrum index (ASI) [16], was better able to capture changes in the quality and appropriateness of antibiotic use in a NICU population than the more commonly used metric, normalized days of therapy [18]. Our prospective, longitudinal case-control study comparing preterm infants with bacteremia to uninfected controls uses the ASI to objectively measure intensity of antibiotic exposures and associations with gut microbial diversity over time, adding to our understanding of the adverse effects and key signatures of antibiotic use on the infant microbiome.

## Methods

### Study design

Eligible infants were admitted to the NICU at Vanderbilt University Medical Center (VUMC) from July 2021 to July 2022, weighed ≤2000 g at birth, and were ≤2 months postnatal age (PNA) at enrollment. We excluded patients with cyanotic congenital heart disease or congenital malformations of the gastrointestinal tract. Stool samples were collected weekly from diapers or syringes of ostomy output into ZYMO DNA/RNA Shield Fecal Collection Tubes and transported to the lab within 24 h. Samples were then aliquoted and frozen at −80 °C until analysis. Prospective weekly stool collection began at birth and continued until discharge or transfer to another hospital.

### Patient population & enrollment

We performed retrospective medical record review for clinical and demographic information. Any infant with a positive blood culture was identified as a bacteremia case. Positive blood cultures with true pathogens were distinguished from possible contaminants, as detailed in Supplemental Methods. Uninfected controls had no culture-proven infections from any specimen source and received ≤5 total days of antibiotics during admission. Controls were matched 1:1 to cases by GA at birth +/−7 days, and PNA at time of stool collection +/−14 days. NEC diagnoses were limited to Modified Bell’s classification stage 2 or greater.

### Sample selection & processing

Each subject had 5–8 longitudinal stool samples selected for analysis; for cases, these included 1–2 samples prior to a bacteremia event and 4–5 after the event. For metagenomic analyses, microbial DNA from stool samples was extracted using ZymoBIOMICS DNA Miniprep Kit (Zymoresearch). DNA-seq libraries were made and sequenced on an Illumina NovaSeq 6000 S4 to an approximate depth of 40 million 2 × 150 bp reads. Negative controls (water, sample collection buffer) and positive controls (with known taxonomic composition) were sequenced concurrently.

### Antibiotic exposure

The ASI [16] was used to quantify intensity of antibiotic exposure for every infant–accounting for spectrum of coverage and duration of therapy. Spectrum scores were assigned to antibiotics according to Gerber et al. [16]. Further details are provided in Supplemental Methods.

### Metagenomic analysis

(1) Preprocessing and quality control of next-generation sequencing (NGS) data: Adapter removal and quality-based trimming of the raw reads were performed using Trimmomatic v0.39 using default parameters [19]. Trimmed reads shorter than 50 nt were discarded. Low complexity reads were discarded using *bbduk* from bbtools [20] with entropy set at 0.7 [21]. (2) Read binning: Reads were mapped to human genome (GRCh38) using *bbmap* from bbtools; the mapped reads were discarded. The remaining reads were used to profile microbial abundance using MetaPhlAn3 [22]. Relative abundances were evaluated at the individual stool/subject level for general patterns and to identify organisms with high abundance, including whether the bacteremia pathogen predominated in the preceding stool sample. The definition for high abundance was ≥30% relative abundance of a single species [9, 14].

### Statistical analysis

We calculated α-diversity, β-diversity, and differential abundance (individual taxa) comparing all cases to controls, and also in a subgroup analysis excluding contaminants and separating cases into those with and without NEC. Statistical analyses were conducted at the species level using Phyloseq [23], Vegan and R version 3.6.3 [24]. The α-diversity analyses used observed species, Shannon, and inverse Simpson indices; for β-diversity, Bray-Curtis and Jaccard indices were used. R package splinectomeR was used for statistical significance in longitudinal microbiome data [25], with the sliding spliner function used to test the data series at defined intervals and whether two groups of interest were significantly different at any point during the time series. Clinical data were compared using the independent *t*-test (for continuous variables) and the chi-square test or Fisher Exact Test (for categorical variables). Statistical significance was defined as a *p*- or *q*-value < 0.05.

### Ethics

This study was approved by the Institutional Review Board at Vanderbilt University with a waiver of informed consent. Study data were collected and managed using REDCap electronic data capture tools hosted at VUMC [26, 27].

## Results

### Microbiological, clinical and demographic characteristics

During the study period, 398 stool samples from 40 cases and 39 controls were selected for whole-metagenome sequencing. Ostomy specimens comprised 10 samples in the cases group. Each infant contributed a mean of 5 longitudinal stool samples (range 1–8). Pathogens for the 40 bacteremia cases included: coagulase negative *Staphylococcus* (CONS) spp. (*n* = 19), *Klebsiella* spp. (*n* = 7), *Staphylococcus aureus* (*n* = 3), *Escherichia coli* (*n* = 2), *Enterococcus* spp.(*n* = 2), *Streptococcus agalactiae* (Group B Streptococcus [GBS], *n* = 2), *Enterobacter cloacae* (*n* = 1), *Citrobacter freundii* (*n* = 1), *Serratia marcescens* (*n* = 1), *Bacillus cereus* (*n* = 1), and an aerobic diphtheroid (*n* = 1). Detailed descriptions of the 40 cases can be found in Supplemental Table 1.

Baseline demographic information is shown in Table 1. Compared to controls, cases had longer NICU admissions (*p* < 0.001). There were no significant differences in maternal factors, including frequency of chorioamnionitis, GBS status, COVID-19 infection, or intrapartum antibiotics. Among the 40 infants with positive blood cultures, 28 (70%) had true pathogens, and 11 (28%) had suspected contaminants (see definition in Supplemental Methods). Medical or surgical NEC occurred in 15 cases (38%) and spontaneous intestinal perforation (SIP) in 2 cases (5%). Bacteremia occurred within 48 h of NEC diagnosis in 6 of the 15 cases (40%).

**Table 1:** Demographic and clinical characteristics.

	Case (*n* = 40)	Control (*n* = 39)	*p*-value
Male sex, *n* (%)	22 (55)	25 (64)	0.68
Gestational age (weeks), mean (SD)	27.1 (22.1)	27.5 (15)	0.29
Birth weight (kg), mean (SD)	0.93 (0.34)	0.96 (0.2)	0.61
Multiple gestation, *n* (%)	9 (23)	4 (10)	0.22
Vaginal birth, *n* (%)	10 (25)	5 (12)	0.35
Hispanic ethnicity, *n* (%)	7 (18)	3 (8)	0.31
Race, *n* (%)			
White	20 (50)	20 (51)	0.9
Black	14 (35)	12 (31)	
Other or unknown	6 (15)	7 (18)	
Mortality, *n* (%)	4 (10)	0 (0)	–
Length of admission in days, mean (SD)	141 (94.5)	77 (26.5)	<0.001
Nutrition/diet by stool sample	(*n* = 383 samples)	(*n* = 249 samples)	
Enteral feeding^a^, *n* stools (% stools)			
Maternal breast milk (MBM)	157 (41)	141 (57)	<0.001
Donor breast milk (DBM)	81 (21)	90 (36)	
Formula	190 (50)	78 (31)	
None/NPO	62 (16)	5 (2)	
Maternal chorioamnionitis^b^, *n* (%)			
Yes	9 (23)	8 (21)	0.98
No	20 (50)	20 (51)	
Unknown	11 (28)	11 (28)	
Maternal antibiotics, *n* (%)			
Any perinatal antibiotics	32 (80)	30 (77)	0.74
None	5 (13)	0 (0)	–
Unknown	3 (8)	9 (23)	–

*P*-values computed using independent t-test for continuous variables, chi-square or Fisher Exact Test for categorical variables with statistical significance defined as *p* <0.05.

^a^Enteral feeding describes all diets received by the subject in the 7 days prior to a collected stool sample. In a given week a subject could have multiple feeding regimens; hence percentages add to >100%; NPO, nothing by mouth.

^b^Chorioamnionitis was based on histologic evaluation of placenta.

Compared to controls, cases had higher mean cumulative ASI at multiple points in PNA and NICU admission, including within the first 7 days of life (Table 2). The most frequently used antibiotics were ampicillin and gentamicin, a regimen used routinely as empiric coverage of early onset sepsis (EOS). Cases were more likely than controls to have received vancomycin (80% vs. 8%, *p* < 0.001) and cefepime (70% vs. 5%, *p* < 0.001).

**Table 2:** Antibiotic exposure among case and control infants.

Cumulative ASI^a^ by subject, mean	Case (*n* = 40)	Control (*n* = 39)	*p*-value
ASI at 7 days of life	22.7	8.4	<0.001
ASI at 30 days of life	94.8	10.8	<0.001
ASI at time of bacteremia	110.6	n/a	
ASI at NICU discharge	252.4	11.4	<0.001
Preceding ASI^b^ by sample, mean	Case (*n* = 199 stools)	Control (*n* = 199 stools)	
ASI in 7 days preceding stool collection	11.3	0.5	0.001
ASI in 14 days preceding stool collection	21.8	1.7	<0.001
Total exposed to antibiotic, *n* (%)^c^	Case (*n* = 40)	Control (*n* = 39)	
Ampicillin	37 (92.5)	27 (69.2)	0.01
Gentamicin	37 (92.5)	26 (66.7)	0.005
Vancomycin	32 (80)	3 (8.0)	<0.001
Cefepime	28 (70)	2 (5.0)	<0.001
Metronidazole	19 (47.5)	2 (5.0)	0.0
Cefotaxime or ceftriaxone	18 (45)	0 (0)	–
Cefazolin	17 (42.5)	0 (0)	–
Piperacillin-tazobactam	11 (27.5)	0 (0)	–

*P*-values calculated using independent t-test for ASI, chi-square or Fisher Exact Test for categorical antibiotic exposures, with statistical significance defined as *p* < 0.05.

^a^Cumulative ASI is the sum of all antibiotics up to that timepoint in the subject’s NICU admission while ^b^preceding ASI is the sum of antibiotics received only during a specific interval prior to individual stool collections.

^c^Total exposed is the number of subjects who received the listed antibiotic at any point during admission. More details including ASI calculations available in supplemental methods.

### Intestinal microbiome diversity and subsequent infection

**Fig. 1:**
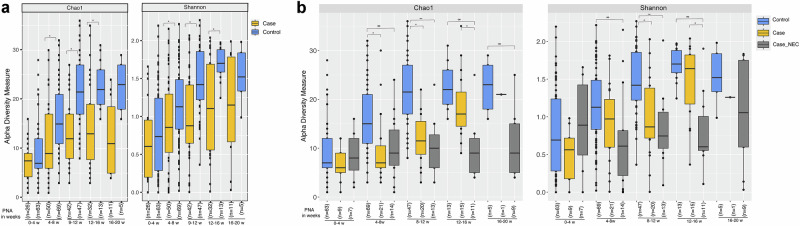
Microbiome α diversity in case vs. control stool samples, grouped by advancing postnatal age in weeks. **a** Microbiome α diversity by Chao1 and Shannon Diversity Indices in case vs. control stool samples, grouped by advancing postnatal age. **b** Case sub-groups separated by history of NEC/SIP diagnosis (Case_NEC) or no NEC diagnosis (Case) compared to controls, with suspected contaminant cases removed; grouped by advancing postnatal age. The *p*-values were computed using the pairwise Wilcoxon test. (* indicates *p*-value < 0.05).

When stool samples were pooled and evaluated by advancing PNA, controls had significantly higher α-diversity (a measure that accounts for the richness and evenness of the different microbial species present in a sample) than cases by both the Chao1 and Shannon Diversity Indices in all samples obtained at a PNA > 4 weeks (Fig. 1a. Corresponding β diversity measures (Principal Coordinate Analyses [PCoA]) are shown in Supplemental Fig. 1. In sub-analysis, we excluded suspected contaminant cases (*n* = 11) and further separated the remaining 29 cases into two subgroups: cases with NEC or SIP (Case_NEC, *n* = 12) and cases with bacteremia who were never diagnosed with NEC or SIP (Case, *n* = 17). After this refinement, we observed that the cases group (without NEC) exhibited significantly lower diversity than controls (Fig. 1b). However, cases with NEC showed more substantial microbiome diversity loss, with significantly lower diversity than cases without NEC and control groups (Fig.1b).

**Fig. 2:**
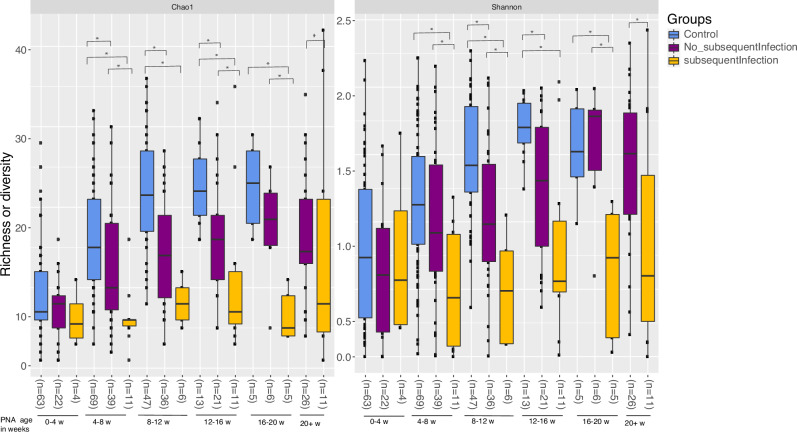
Microbiome α diversity of cases with subsequent infection, cases with no subsequent infection, and uninfected controls, grouped by advancing postnatal age. Microbiome α diversity of cases with subsequent infection, cases with no subsequent infection, and non-infected controls, grouped by advancing postnatal age. Subsequent infection after bacteremia was defined as having a culture confirmed bacterial infection, necrotizing enterocolitis, or spontaneous intestinal perforation occurring ≥2 weeks after the blood culture date (*n* = 8). The *p*-values were computed using the pairwise Wilcoxon test. (* indicates *p*-value < 0.05).

In additional analysis of all 40 cases, cases that developed subsequent infections after the index bacteremia event (*n* = 8) were compared to cases with no further infectious complications after the bacteremia event (*n* = 32) and to controls (Fig. 2). With advancing PNA, stool samples from the cases with no subsequent infections after the single bacteremia event had recovering α-diversity to levels comparable to the non-infected controls, while samples from those cases with subsequent infections failed to recover and showed stagnation of α-diversity up to 20 weeks PNA (Fig. 2). The most common subsequent infections in those cases included respiratory tract, urinary tract, and NEC. Compared to cases with no subsequent infection, cases with subsequent infection had higher mean cumulative ASIs at the time of positive blood culture (118.5 v. 108.6, *p* = 0.864) and at NICU discharge (430.3 v. 207.9, *p* = 0.074), though these differences were not statistically significant. Corresponding PCoA comparing β-diversity of these groups is shown in Supplementary Fig. 2.

To minimize the impact of very recent or concurrent antibiotic use, with the suspicion that these exposures might more strongly but transiently influence a sample’s microbial composition, we analyzed samples with a 14-day preceding ASI of 0. Among these samples from subjects without recent antibiotic exposure, a significantly lower α-diversity persisted in PNA matched cases compared to controls (Supplementary Fig. 3).

### Anaerobic and *Enterococcus* spp. abundance

**Fig. 3:**
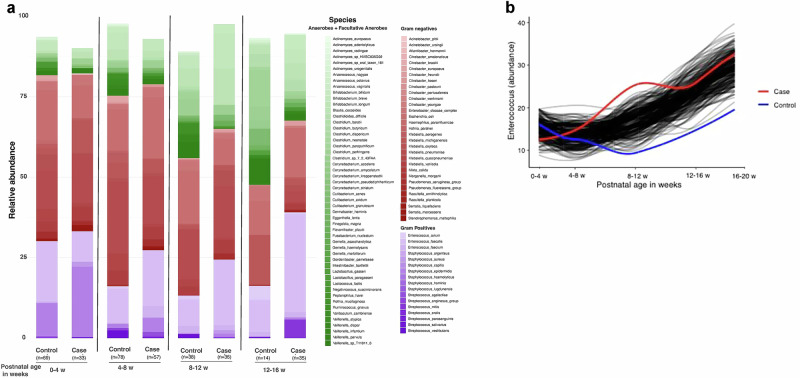
Relative abundance of organisms in case vs. control stool samples. **a** Relative abundance of organisms in case vs. control stool samples, grouped by advancing postnatal age. Green = obligate and facultative anaerobes, brown = gram negative bacteria, purple = gram positive bacteria. **b** Permuted spline tests for statistical significance in longitudinal microbiome data depicting *Enterococcus* (genus) abundance over advancing postnatal age in weeks (x-axis), in total case (group spline in red) vs. control samples (group spline in blue, *p*-value = 0.01).

The average relative abundance of gram negative (GN), gram positive (GP), and anaerobic organisms was initially comparable for cases and controls in pooled samples at ≤4 weeks PNA, with high GN bacteria and low anaerobe prevalence (Fig. 3a). As PNA advanced, control stools had increasing abundance of anaerobes –for example, in pooled samples at 13–16 weeks PNA, anaerobes comprised 50% of species abundance. By comparison, case stools demonstrated increasing *Enterococcus* spp. abundance. At 13–16 weeks PNA, pooled case stools were >25% *Enterococcus* spp. abundance and had lower total anaerobe presence (25%). When cases were separated into those with and without NEC, *Enterococcus* spp abundance was higher by 12 weeks PNA in cases with NEC compared to cases without NEC and controls (Supplemental Fig. 5). When evaluated at the individual subject level, there was high *Enterococcus* spp. abundance in more cases (*n* = 24) than controls (*n* = 13, *p* = 0.02). Among individual samples with high *Enterococcus* abundance, the mean infant age at the time of sample collection was significantly younger in controls (PNA: 6.4 weeks in controls vs. 13.3 weeks in cases, *p* < 0.01).

We performed sub-analysis using the sliding spliner function in splinectomeR [25] to investigate whether the observed differences in *Enterococcus* spp. relative abundance were significant over time. Comparable species abundances observed during the first 4 weeks of life were confirmed to diverge significantly in *Enterococcus* spp. abundance in the temporal model of cases vs controls (Fig. 3b), with significantly higher *Enterococcus* spp. in cases at 8–16 weeks PNA (*p* = 0.01). Similar trends were found when samples were grouped by antibiotic use, including samples stratified by 14-day preceding ASI (Supplementary Fig. 4a–c).

### Clinical features associated with high stool Enterococcus spp. abundance

Sub-analysis of cases with high *Enterococcus* spp. abundance (*n* = 24; defined as ≥1 stool with ≥30% *Enterococcus* spp.) compared to cases without high *Enterococcus* spp. abundance (*n* = 16) investigated clinical factors associated with this microbiome feature. Those cases with high *Enterococcus* abundance were significantly more likely to have multiple infectious episodes during admission (*n* = 15, 63%) than those without high *Enterococcus* abundance (*n* = 3, 19%) (*p* < 0.01). Though not significantly different, cases with high *Enterococcus* abundance also tended to have longer NICU admissions (161 v. 110 days, *p* = 0.09), more NEC or SIP diagnoses (13 v. 4, *p* = 0.1), and higher cumulative ASI (289 v. 197, *p* = 0.20) and 14-day preceding ASI (27 v. 20, *p* = 0.22) than cases without high *Enterococcus* abundance. Among controls, no significant differences were found in GA at birth, birth weight, length of admission, or cumulative ASI at discharge in controls with or without high *Enterococcus* stool abundance.

## Discussion

Using whole-metagenome sequencing of longitudinal stool samples from preterm infants we determined that, compared to uninfected controls, infants with bacteremia had significant stagnation in their gut microbiome diversity. This persisted in cases with isolated bacteremia but was more strongly potentiated, and longer lasting, in cases with both bacteremia and a NEC/SIP diagnosis. Specifically, cases had higher *Enterococcus* spp. and lower anaerobe abundance, and these patterns were associated with recurrent infectious complications. Notably, there was no significant difference in microbiome diversity or relative abundance between control samples from 0 to 4 weeks PNA, suggesting that the infants were similar in gut microbiome compositions at the time of study inclusion.

The ASI scoring allowed objective comparison by subject and by individual stool sample, and helped to illustrate in sub-analysis of samples with 14-day preceding ASI = 0 that the microbiome differences observed between case and control stools were not transient nor dynamic responses to current treatment (in which case we would expect the diversity differences to disappear or lose significance in samples taken a few weeks after stopping antibiotics). The cumulative effect of repeated exposure to antibiotics may have a more lasting influence on the infant gut microbiome than currently appreciated. In addition to the need to investigate the long-term durability of microbiome alterations attributable to antibiotics, these results could also illustrate that many other clinical factors drive dysbiosis, including inflammation, diet, and infection.

While bacteremia cases that did not have subsequent infection following their initial bloodstream infection had recovery of microbiome diversity at older PNAs, mimicking their age-matched controls, the subset of cases who developed subsequent infections had pervasive differences in gut microbiome diversity up to 20 weeks PNA. It is unclear whether microbiome changes are merely surrogates for or primary drivers of repeated infection. Cases with NEC/SIP diagnosis similarly showed more severe and lasting α-diversity stagnation and likely have compounding risk factors at play, including but not limited to: local gut wall inflammation or damage, longer interruptions to enteral feeding, and extended time on broad-spectrum antibiotics (vs targeted antibiotics for isolated bacteremia or line infection). Future research is needed to understand why some neonates experience only a single bacteremia event, while others follow a trajectory of recurrent infection and clinical decompensation over prolonged NICU admissions.

Comparison of relative species abundance among case and control samples revealed two notable trends in microbial compositions. First, control samples showed a consistent increase in anaerobe abundance with advancing age—a marker of healthy gut maturation with many beneficial commensals in this category [5, 12]. Second, while the abundance of GN and GP organisms decreased over time in controls, cases demonstrated increasing *Enterococcus* spp. abundance, with significantly more cases having high *Enterococcus* spp. abundance in ≥1 stool sample. Additionally, the control stools with high *Enterococcus* spp. abundance were collected from infants at significantly younger PNA ages on average compared to case stools, leading us to suspect that the high *Enterococcus* spp. abundance at advanced PNA in cases reflects delayed maturation of the premature infant gut.

An increase in multiple infectious episodes among cases that had high *Enterococcus* spp. in their stools argues for the clinical significance of this microbiome signature. Whether *Enterococcus* spp. abundance drives the next infectious episode, develops as a result of serial antibiotic exposures, or persists secondary to ongoing gut inflammation remains elusive. These results add to a growing body of literature suggesting that *Enterococcus* spp. overabundance signals gut microbiome dysbiosis and poor clinical outcomes in high risk patients [8, 9], including neonates.

We recognize several limitations of this study. It is observational, descriptive, and cannot draw conclusions about causation. Complicated antibiotic histories make it challenging to associate specific microbiome changes to any one antibiotic or duration of treatment. We were unable to assess durability of microbiome changes beyond 5 months PNA as infants were typically discharged by this time. Other known factors that impact the microbiome, such as nutrition, could not be independently evaluated in our study design, largely because of frequent changes in enteral and parenteral feeding at times of illness. However, we matched cases and controls by GA at birth and PNA with the intention that these act as surrogates for other clinical factors (e.g., the initial diet and feeding source at birth corresponded in matched cases and controls as both groups followed the same age-based enteral feeding advancement protocols). Some samples from NEC (+/−SIP) patients were derived from ostomy output, which typically originates from the small intestine. These samples may differ from stool in microbial composition, limiting our ability to assess site-specific diversity across intestinal regions such as the ileum, jejunum, and colon. As a single-center study, our findings may reflect species-level microbial abundances that are unique to our NICU environment and patient population and may not be generalizable to other clinical settings. Despite these limitations, strengths of our study include our use of the ASI, which enabled us to quantify complicated antibiotic exposures (rather than the binary, yes/no metric of antibiotic exposure used in previous research) and increase applicability of our findings to complex NICU populations.

Our findings have important clinical implications and suggest areas for future research. In hematopoietic stem cell transplant patients, literature indicating that high *Enterococcus* spp. abundance and other microbiome alterations contribute to the incidence of GvHD has led to changes in clinical management [28] and clinical trials to investigate antibiotic regimens that minimize harm to healthy gut commensals [29]. As signatures of intestinal microbiome dysbiosis in neonates emerge, a similar proactive approach could be taken in the NICU where prescribers more strongly consider the microbiome impact of different antibiotic choices. Our study indicates an association between low anaerobes, high *Enterococcus* spp. abundance and poor outcomes, including recurrent infection in preterm infants. Further investigations in cohorts with larger sample sizes are needed to confirm and explain the basis of these findings. Antibiotics that preferentially target anaerobic bacteria or enable overgrowth of *Enterococcus* spp. may be important targets for antibiotic stewardship interventions.

## Supplementary information

Supplemental Materials

## Data Availability

Upon publication, raw metagenomic sequencing data will be available in the Sequence Read Archive (SRA) under BioProject identifier SUB14269517.
