# Supplementary material for: Associations between antibiotic exposure intensity, intestinal microbiome perturbations, and outcomes in premature neonates with bacteremia
Source: J Perinatol. 2025 Jun 9;45(7):986–92. doi: 10.1038/s41372-025-02330-0 (PMC12316594; doi:10.1038/s41372-025-02330-0)

Supplemental Methods

<u>Definitions:</u>

**Early onset sepsis (EOS)**	a positive blood culture at ≤7 days of life (DOL)
**Empiric treatment/ culture negative EOS**	clinical episode treated with ≥5 days of antibiotics at ≤7 DOL with a negative blood culture and no alternative or culture-proven infection
**Late onset sepsis (LOS)**	a positive blood culture at >7 DOL
**Empiric treatment/ culture negative LOS**	clinical episode treated with ≥5 days of antibiotics begun at >7 DOL and after sepsis evaluation (ie. cultures and infection labs), with a negative blood culture and no alternative or culture-proven infection
**True pathogen**	*Staphylococcus aureus, Streptococcus agalactiae* or a member of the Enterobacterales order in any positive blood culture or a typical commensal organism (such as coagulase negative *Staphylococcus* spp. (CONS)) if the isolate grew in >1 serial blood culture and/or was treated with ≥5 days of antibiotics
**Suspected contaminant**	a typical commensal organism (ie aerobic diphtheroid, CONS) which grew in a single blood culture and was treated with <5 days of antibiotics
**Subsequent infections** (after bacteremia)	any other culture-proven bacterial infection, necrotizing enterocolitis or spontaneous intestinal perforation ≥2 weeks after positive blood culture
**Multiple infections** (during admission)	any other culture-proven bacterial infection, necrotizing enterocolitis, or spontaneous intestinal perforation ≥2 weeks before or after positive blood culture
**Cumulative Antibiotic Spectrum Index (ASI)**	a numeric score/total of all antibiotics received by the subject from birth until the indicated timepoint (ie 30 DOL, bacteremia event, discharge)
**Preceding ASI**	a numeric score/total of only the antibiotics received by the subject during a key interval of time preceding stool collection (ie 7 or 14 days prior to stool collection)

Antibiotic Spectrum Index (ASI):

For each antibiotic the infant received, the appropriate spectrum score was multiplied by the total days of therapy to yield the ASI. If infants received multiple antibiotics, the total ASI was a summation of the spectrum score[15] multiplied by the duration of therapy for each antibiotic.

ASI = (spectrum score[1] of antibiotic A) × (duration in days of antibiotic A)

Cumulative ASI = (antibiotic A spectrum × antibiotic A duration) + (antibiotic B spectrum × antibiotic B duration) + (antibiotic C spectrum × antibiotic C duration)

Example: Cumulative ASI for a 36-hour neonatal sepsis “rule out” at birth

Ampicillin spectrum score: 2; duration: 2 days (dosed q6h over 36 hours)

Gentamicin spectrum score: 5; duration: 1 day (dosed q48h, given once)

Cumulative ASI = (2 × 2) + (5 × 1) = 9

In order to quantify recent (rather than cumulative or total) antibiotic exposure, “preceding ASI” was also calculated and included only antibiotics received during the 7- or 14-day time period preceding stool collection. This was calculated using the same equation as cumulative ASI, but restricted to antibiotics given on days -7 to 0 before stool collection (for 7-day preceding ASI) or -14 to 0 days (for 14-day preceding ASI). An antibiotic course which extended beyond the 7- or 14-day period received points only for the days falling within the window.

[1]The spectrum score/numeric value assigned to each antibiotic followed those published in Table 1: Antibiotic Spectrum Index Matrix by Gerber et al. (15)

Supplemental Table 1. Clinical details for 40 neonatal bacteremia cases.

**Case:**	Sex	GA	BW	PNA	Organism	Diagnosis	Treatment	NEC/SIP	Outcome	Stools
1	M	26.2	0.50	14.4	*S. epidermidis*	TP	V x 10d	Med**	alive	7
2	F	22.2	0.42	27.6	*S. epidermidis*	SC	V/G/C^{4} x 2d	Med**	alive	6
3	F	27.4	1.055	0.7	*Aerobic diphtheroid*	SC	A/G x 2d	None	alive	6
4	F	24.3	0.68	4.3	*S. epidermidis*	TP	V x 5d, C^{4}/F x 11d	Med***	alive	6
5	F	29.2	1.30	1.7	*E. coli*	TP	C4/F x 25d	Surg***	dead	7
6	M	22.1	0.51	10.6	*K. pneumoniae*	TP	C^{4} x 5d, C^{3} x18d	Med**	alive	6
7	F	27.4	1.01	5.4	*S. hominis*	SC	None	None	alive	5
8	M	24.3	0.70	6.0	*K. aerogenes*	TP	C^{4} x 21d	None	alive	6
9	F	24.1	0.68	24.9	*S. epidermidis*	SC	V/C^{4} x2d, C^{3} x4d	Med**	alive	6
10	M	27.3	0.655	7.1	*S. epidermidis*	SC	V x 3d	None	alive	6
11	F	25.5	0.37	26.3	*S. epidermidis*	TP	V/C^{4} x 6d	None	alive	5
12	M	31.5	1.375	0.9	*S. aureus*	TP	V/G x 13d, C^{5}/R x 4d^	None	dead	1
13	M	27	1.05	0.9	*S. epidermidis*	TP	V/G/F x 15d	SIP***	alive	6
14	M	24.5	0.785	5.6	*S. agalactiae*	TP	C^{4} x 22d	Med*	alive	6
15	F	24.3	0.83	1.6	*K. oxytoca*	TP	V/C^{4} x 3d, C^{3} x 10d	None	alive	6
16	M	27.5	1.09	1.3	*S. aureus*	TP	N x 8d	None	alive	3
17	F	29.2	0.82	8.0	*S. agalactiae*	TP	A x 14d	None	alive	5
18	M	31	0.97	21.3	*S. capitis*	SC	V/G x 3d	Med**	alive	6
19	F	25.3	0.745	1.0	*S. epidermidis*	SC	None	None	alive	6
20	M	31.2	1.55	3.0	*K. pneumoniae*	TP	C/F x 14d	Med**	alive	3
21	M	25.1	0.84	22.9	*S. capitis*	TP	V/C^{3} x 2d, C^{4} x 8d	Surg**	alive	5
22	F	24.4	0.72	12.3	*E. cloacae*	TP	C^{4} x 10d	None	alive	5
23	F	24.5	0.75	1.0	*K. pneumoniae*	TP	C^{3}/F x 23d	Med***	alive	6
24	F	26.1	1.205	11.7	*C. freundii*	TP	C^{4} x 15d	None	alive	6
25	M	25.5	0.58	18.6	*S. aureus*	TP	V/R x 46d	None	alive	5
26	M	29.1	1.12	0.0	*CONS, no ID*	SC	V x 4d	Surg*	alive	5
27	F	26.3	0.76	10.3	*S. marcescens*	TP	C^{4} x 14d	None	alive	5
28	M	32.1	1.6	3.0	*E. coli*	TP	C^{3} x 21d, F x 8d	None	dead	1
29	F	24	0.64	3.6	*S. capitis*	SC	V x 3d	None	alive	6
30	M	33.6	1.69	12.0	*S. epidermidis*	TP	V x 7d	None	alive	5
31	F	28.1	0.91	2.4	*S. hominis*	TP	V x 6d	None	alive	6
32	M	29.2	0.935	4.4	*B. cereus*	SC	A/G x 2d	None	alive	6

33	F	25	0.67	3.4	*K. pneumoniae*	TP	C^4/F x 15d	Med***	alive	6
34	F	25	0.682	3.4	*S. epidermidis*	TP	V x 9d	None	alive	4
35	M	34.6	1.46	7.0	*E. faecalis*	TP	A/C^3 x 11d	None	alive	4
36	M	32.5	1.39	2.0	*E. durans*	TP	A/G x 11d	None	alive	5
37	M	24.6	0.945	9.6	*S. capitis*	TP	V x 10d	SIP**	alive	5
38	M	22.2	0.55	6.3	*S. capitis*	TP	V x 9d, A x 5d	None	alive	2
39	M	26.4	0.97	2.1	*K. oxytoca*	TP	None^	Surg***	dead	2
40	M	29.5	1.53	1.3	*S. haemolyticus*	SC	V x 3d	Med***	alive	2

GA= gestational age at birth in weeks.days; BW= birth weight in kilograms; PNA= postnatal age in weeks at the time of bacteremia; TP= true pathogen, treated as true bacteremia event by clinical team; SC= suspected contaminant due to organism, <5 days of antibiotics which provide appropriate coverage of the organism, and growth in ≤1 serial blood culture; Treatment is simplified to the final regimen/most directed antimicrobials, V (vancomycin), G (gentamicin), C^3 (cefotaxime or ceftriaxone), C^4 (cefepime), C^5 (ceftaroline) A (ampicillin), F (metronidazole), R (rifampin), N (nafcillin); ^ indicates treatment was incomplete due to patient demise; NEC= necrotizing enterocolitis, SIP= spontaneous intestinal perforation, Med= medical NEC, Surg= surgical NEC; NEC/SIP timing= *** NEC/SIP within 48 hours of bacteremia, ** NEC/SIP before bacteremia, * bacteremia before NEC/SIP; Outcome= vital status at time of hospital discharge from NICU; Stools= number of stools included in the final analysis for each case

Supplemental Table 2. Clinical features of cases and controls

	Case (n=40)	Control (n=39)
**Underlying medical conditions, n (%)**		
Non-cyanotic congenital heart disease[1]	11 (28)	12 (31)
Respiratory distress syndrome	40 (100)	38 (97)
Genetic/chromosomal abnormality	4 (10)	1 (3)
Urogenital abnormality	6 (15)	1 (3)
Central nervous system disease[2]	20 (50)	12 (31)
Ear, nose, or throat abnormality	1 (3)	1 (3)
**Infectious diagnoses, n (%)**		
Early onset sepsis, ≤7 days of life		
Empiric treatment/culture negative[3]	8 (20)	1 (3)
Culture positive, pathogen	3 (8)	0 (0)
Culture positive, suspected contaminant	2 (5)	0 (0)
Late onset sepsis, >7 days of life		
Empiric treatment/culture negative[3]	7 (18)	1 (3)
Culture positive, pathogen	25 (63)	0 (0)
Culture positive, suspected contaminant	10 (25)	0 (0)
Medical necrotizing enterocolitis (NEC)[4]	11 (28)	0 (0)
Surgical necrotizing enterocolitis (NEC)[5]	4 (10)	0 (0)
Spontaneous intestinal perforation (SIP)[6]	2 (5)	0 (0)
Respiratory decompensation[7]	15 (38)	4 (10)
Sepsis evaluation +/- antibiotics	22 (55)	16 (41)
**Culture positive infections, n events/n infants**		
Central Nervous System (meningitis)	2 / 2	0
Urinary tract infection	8 / 8	0
Respiratory tract (tracheitis, pneumonia)	24 / 10	0
Intra-abdominal (abscess, peritonitis)	2 / 2	0
Other (wound, conjunctivitis)	3 / 2	0
**Procedures[8], n infants**		
Any surgical procedure	21	0

GI (bowel resection, ostomy, G-tube)	14	0
ENT (tracheostomy, airway injections)	3	0
CNS (subgaleal or VP shunt)	3	0
Cardiac (PDA device, heart catheterization)	8	0
Urogenital (circumcision, renal stents)	2	0
Other (eye, MMC repair)	2	0

[1]Includes abnormal echocardiogram or cardiac procedure requiring cardiology follow up at discharge. [2]Includes intraventricular hemorrhage, ventriculomegaly, congenital abnormality of brain or spinal cord. [3]Empiric treatment for early or late onset sepsis did not meet case definition; rather, some cases had separate illness episodes which were treated as culture negative sepsis. [4]Timing: 5 before (≥30 days), 5 with (±7 days of positive blood culture), and 1 after bacteremia (≥30 days). [5]Timing: 1 before, 2 with, and 1 after bacteremia. [6]Timing: 1 before, 1 with bacteremia. [7]Defined as escalation in mode of O^2 delivery excluding a return to prior stable settings after failed wean attempt. [8]Of 41 procedures, 20% (8) occurred in the 30 days preceding bacteremia, 17% (7) in the 30 days following bacteremia. VP, ventriculoperitoneal; PDA, patent ductus arteriosus; MMC, meningomyelocele

Supplemental Table 3. Clinical characteristics of cases with high *Enterococcus* spp. abundant stool vs none

**Clinical factors:**	High abundance[1] (n=24)	No abundance (n=16)	p-value
Mean gestational age	26.9	27.3	0.72
Mean birth weight (kg)	0.89	0.98	0.42
Mean length of admission (days)	161	110	0.09
Mortality, n subjects (%)	2 (8)	2 (13)	1.0
Mean PNA at bacteremia (weeks)	8.6	6.5	0.40
Subsequent infections[2], n subjects (%)	7 (29)	1 (6)	0.11
Multiple infections[3], n subjects (%)	15 (63)	3 (19)	<0.01
NEC or SIP diagnosis, n subjects (%)	13 (54)	4 (25)	0.1
Cumulative ASI at discharge, mean	289	197	0.20
Preceding 14-day ASI, mean	27	20	0.22

[1]High *Enterococcus* spp. abundance was defined as any case with ≥1 stool with ≥30% *Enterococcus* spp. relative abundance. [2]Subsequent infection defined as having a culture confirmed bacterial infection, necrotizing enterocolitis, or spontaneous intestinal perforation occurring ≥2 weeks after the blood culture date. [3]Multiple infections defined as having any other culture confirmed infection, necrotizing enterocolitis (NEC), or spontaneous intestinal perforation (SIP) ≥2 weeks before or after the blood culture date. P-values calculated using independent *t*-test for continuous variables or Fisher Exact Test for categorical variables, with statistical significance defined as $p<0.05$.

Figure Legends

Supplemental Figure 1. Microbiome β diversity in cases vs. controls. Represented with Principal Coordinate Analyses of stool samples clustered in 4 week increments of advancing postnatal ages.

Supplemental Figure 2. Microbiome β diversity for cases with subsequent infections after index bacteremia vs. cases without subsequent infections vs. non-infected controls. Represented with Principal Coordinates Analyses at advancing postnatal ages.

Supplemental Figure 3. Microbiome α diversity of case vs. control samples in subjects that received no antibiotic in the 14 days prior to stool collection. All samples collected had 14-day preceding ASI = 0 and are grouped by advancing postnatal age. P-values computed using pairwise Wilcoxon test. (* indicates p-value <0.05)

Supplemental Figure 4. Permuted spline tests for statistical significance in longitudinal microbiome data depicting *Enterococcus* (genus) abundance in grouped samples over advancing postnatal age (x-axis). Stool samples with (a) any prior (red) vs. no antibiotics ever (blue, p-value = 0.01); (b) 14-day preceding ASI 1 to ≤ 35 (blue) vs. 0 (red, p-value = 0.01); (c) 14-day ASI 1 to ≤ 35 (red) vs. ≥ 36 (blue). Differences observed in 4c are not statistically significant.

Supplemental Figure 5. Relative abundance of organisms in stool samples from cases with or without NEC vs. controls, excluding contaminants. Samples are from cases without NEC (Case), cases with NEC (Case_NEC), and controls, grouped by advancing postnatal age. Green = obligate and facultative anaerobes, brown= gram negative bacteria, purple = gram positive bacteria.

Supplemental Figure 1

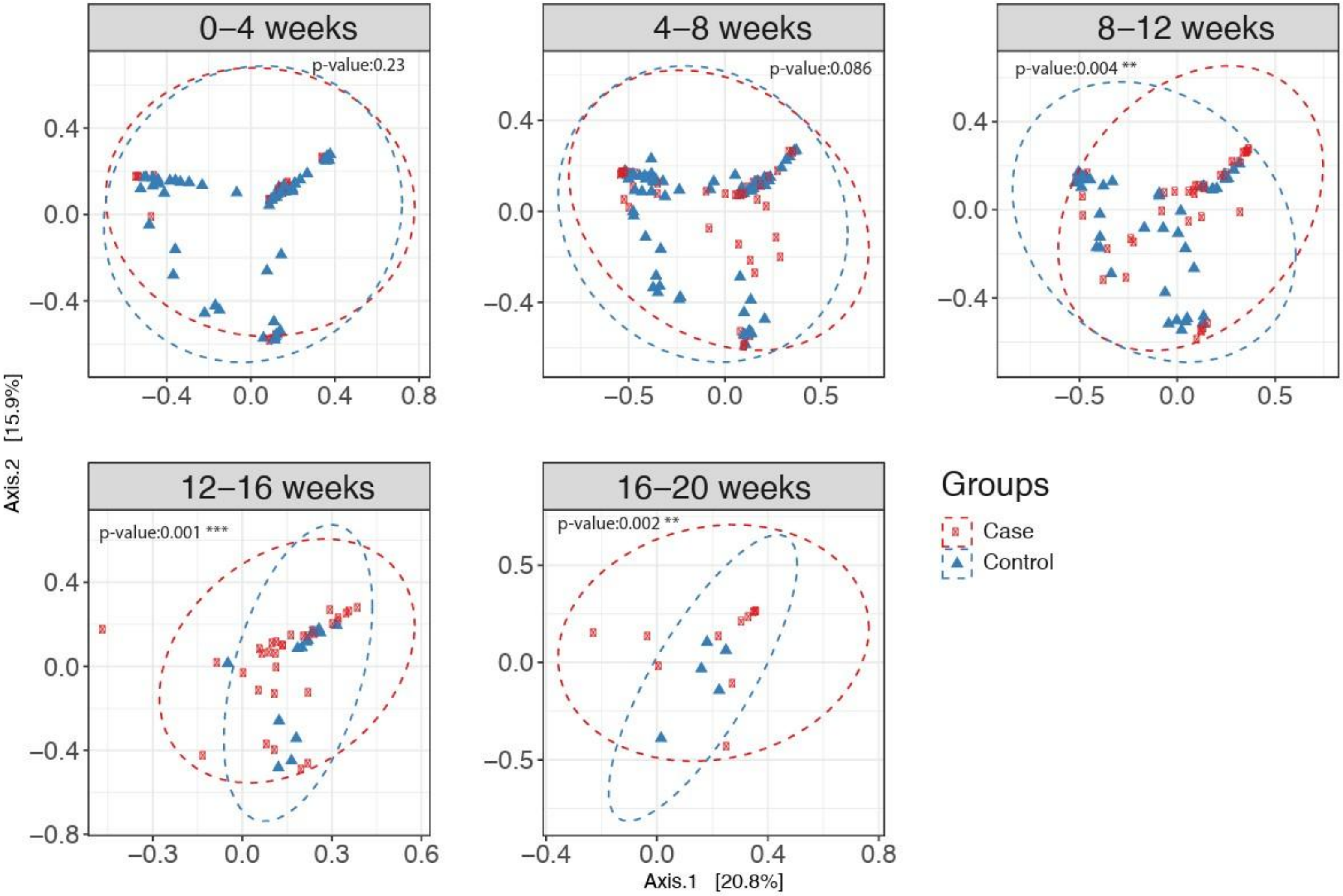

Supplemental Figure 2

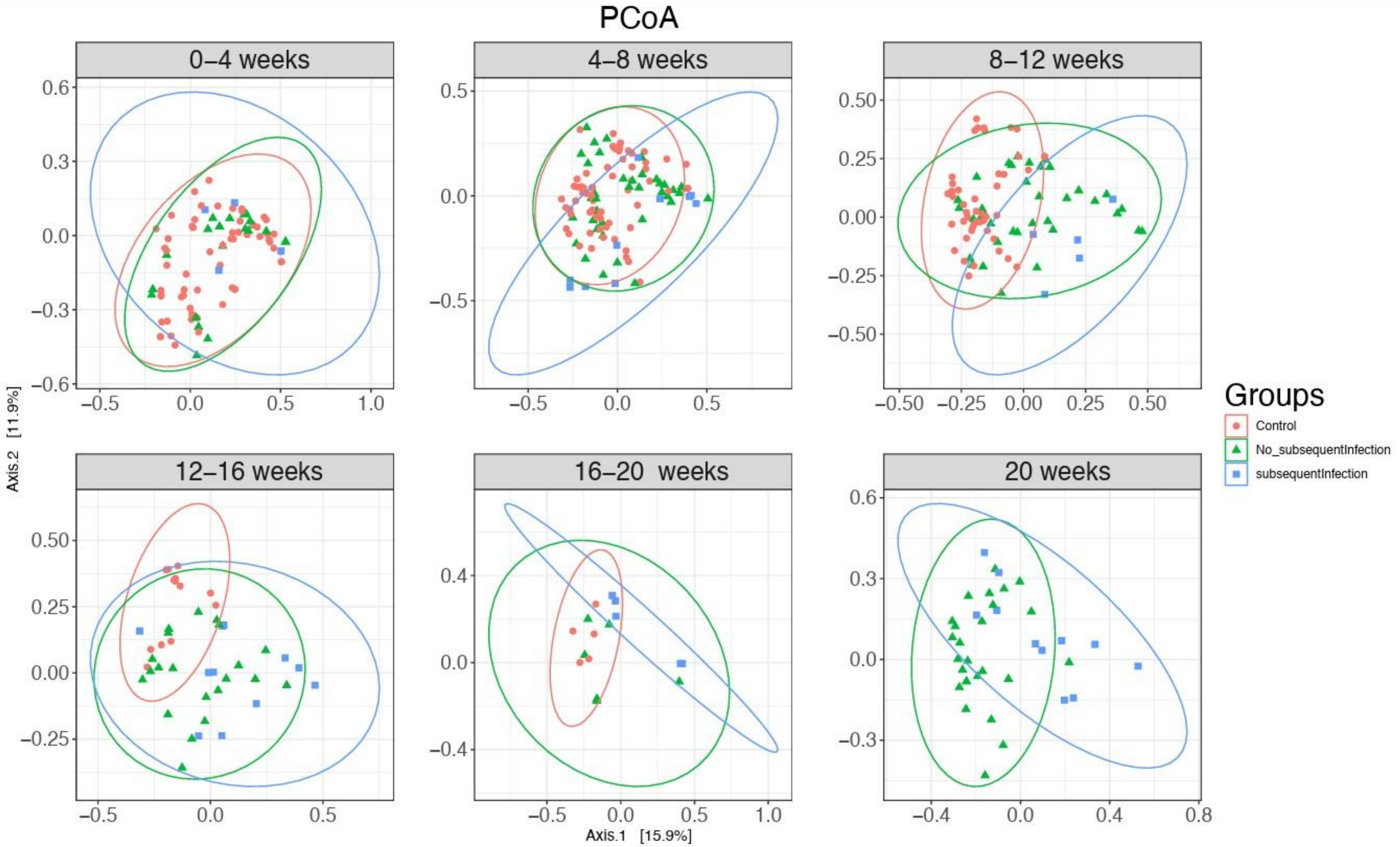

Supplemental Figure 3

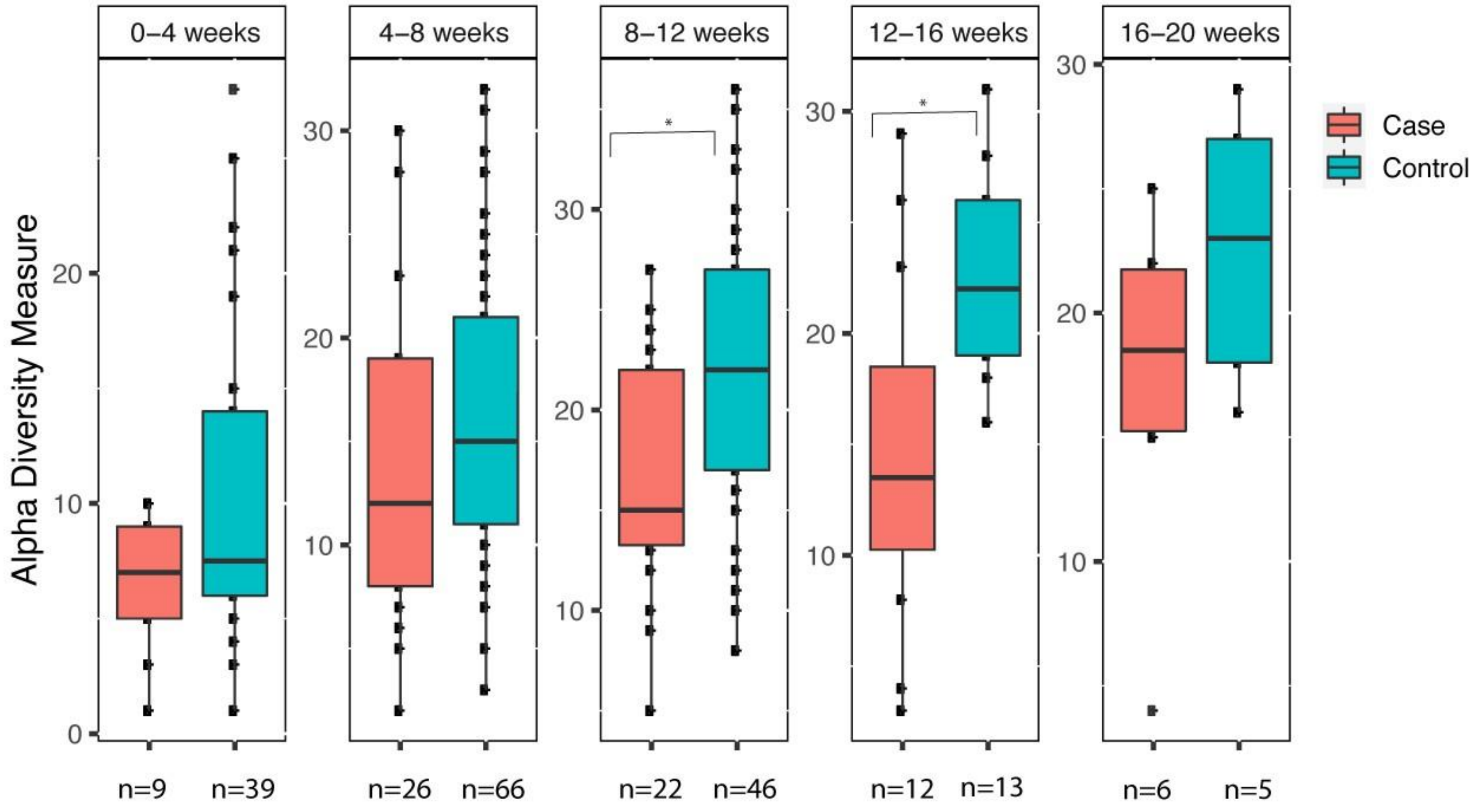

Supplemental Figure 4A

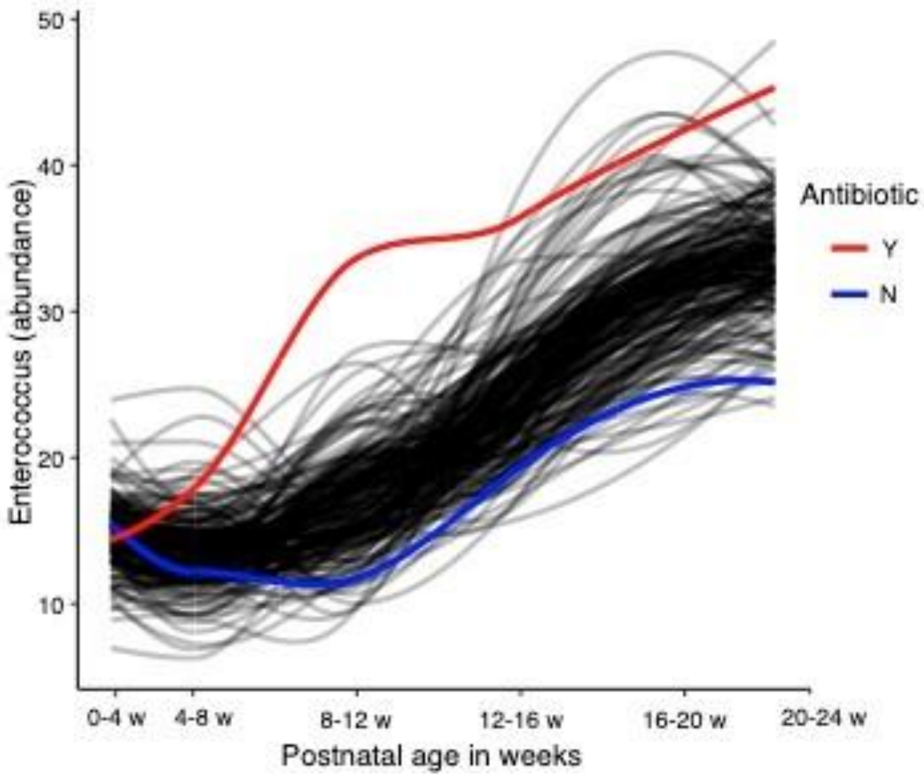

Supplemental Figure 4B

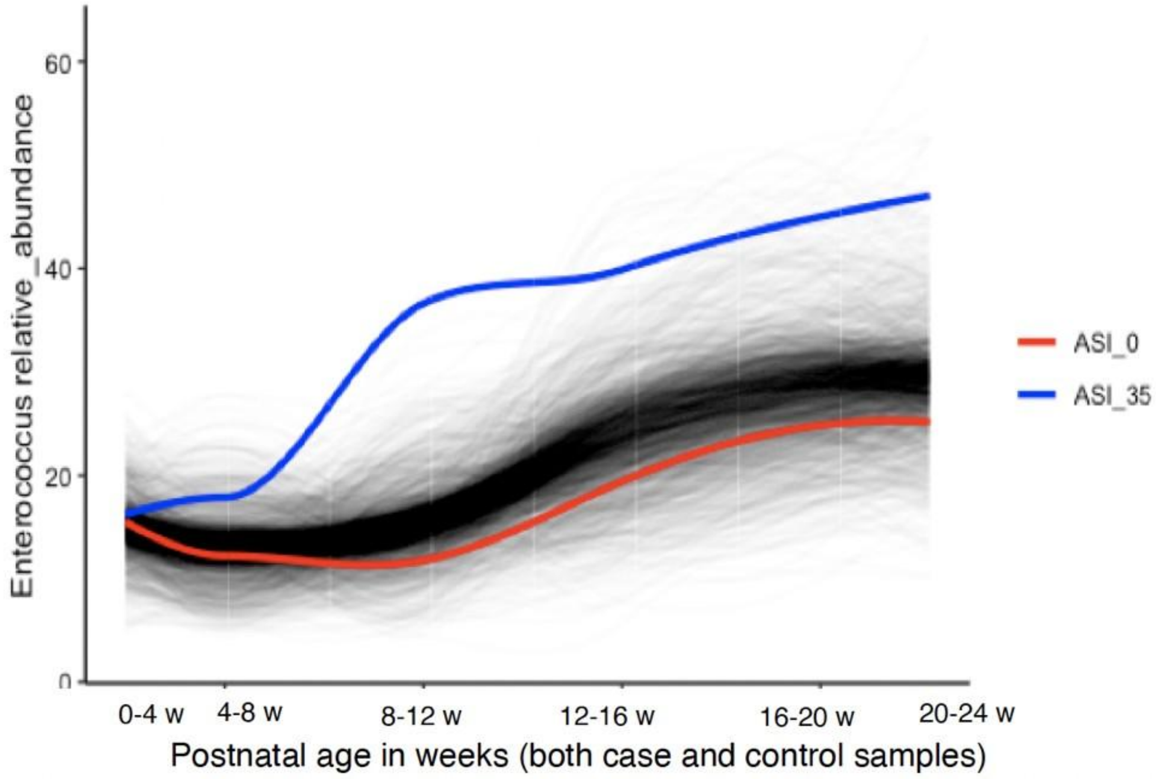

Supplemental Figure 4C

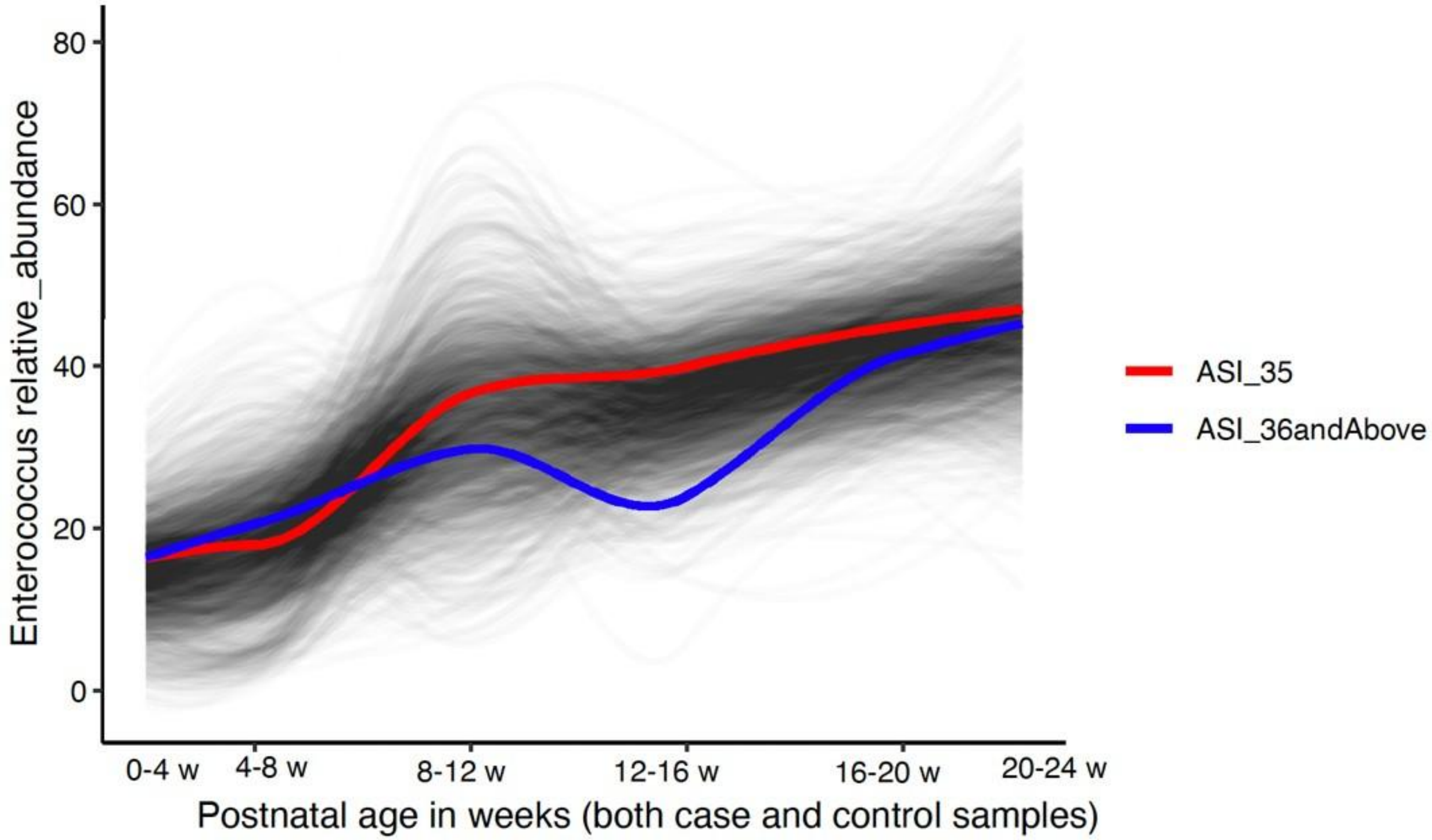

Supplemental Figure 5

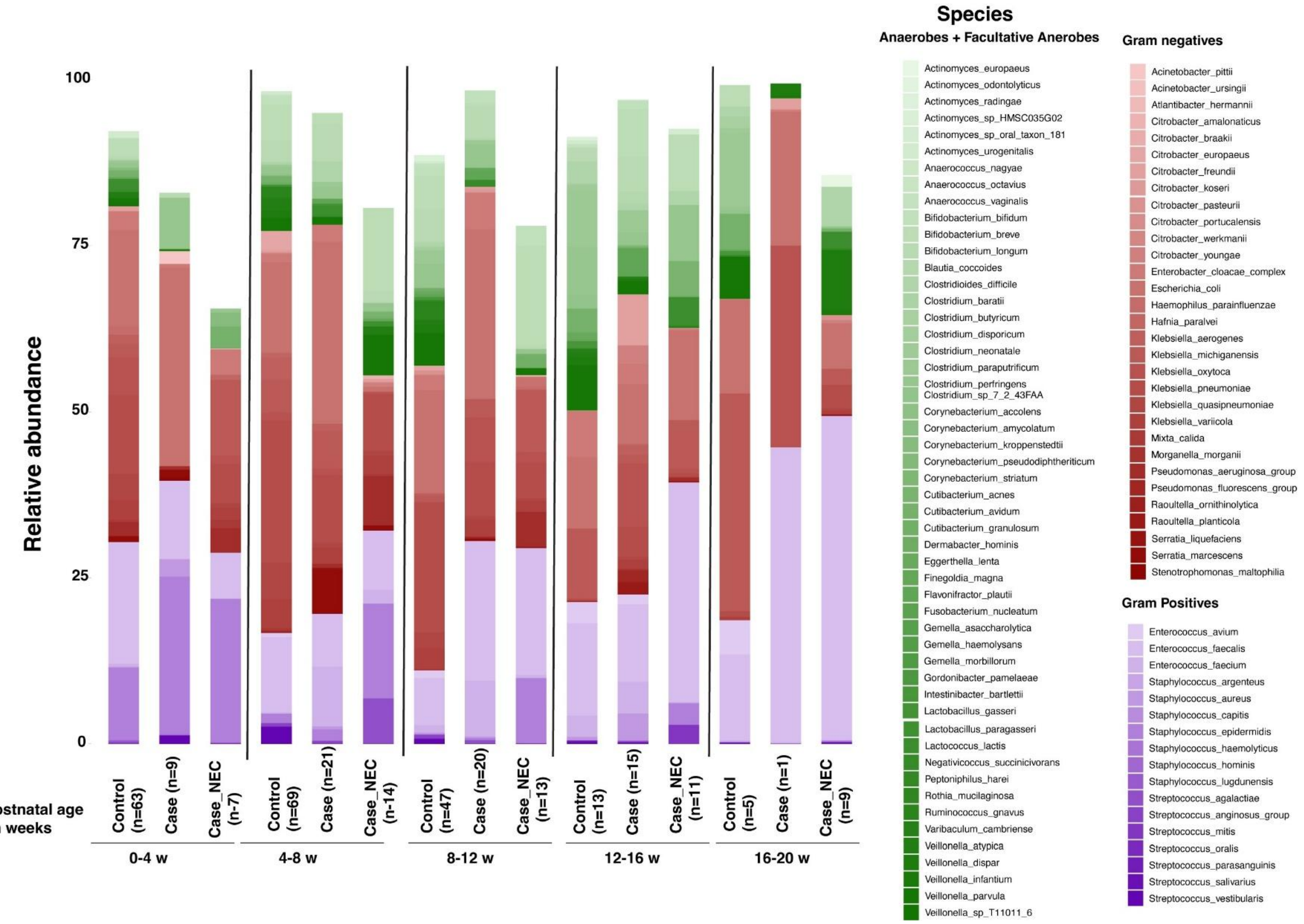

Supplement: Supplementary file 1 — Supplemental Materials [file 41372_2025_2330_MOESM1_ESM.pdf]
